# Exploring the role of long noncoding RNAs in autophagy and cuproptosis processes via immune pathways in head and neck squamous carcinoma: A systematic review of the literature

**DOI:** 10.1097/MD.0000000000039335

**Published:** 2024-08-23

**Authors:** Yao Xiao, Qianrong Li, Yan Li

**Affiliations:** a The Second Affiliated Hospital of Heilongjiang University of Chinese Medicine, Haerbin, Heilongjiang Province, China; b The First Affiliated Hospital of Heilongjiang University of Chinese Medicine, Haerbin, Heilongjiang Province, China.

**Keywords:** autophagy, cuproptosis-related, head and neck squamous cell carcinoma, long noncoding RNA

## Abstract

Cuproptosis, a copper-dependent programmed cell death process, holds promise for controlling cell death in tumor cells. Autophagy, a fundamental cellular process, has been linked to various aspects of cancer, such as proliferation, migration, and drug resistance. This research is centered on the investigation of autophagy- and cuproptosis-related long noncoding RNAs (lncRNAs) and the establishment of a prognostic model for head and neck squamous cell carcinoma. RNA sequencing data from head and neck squamous cell carcinoma patients in The Cancer Genome Atlas database identified cuproptosis-related lncRNAs via Pearson analysis. Patients were divided into training and testing sets. A prognostic model developed in the training set using univariate-least absolute shrinkage and selection operator (Lasso) and multivariate Cox regression was tested for accuracy. Kaplan–Meier analysis showed high-risk patients had poorer outcomes. Cox regression confirmed the model’s risk score as an independent prognostic indicator, with receiver operating characteristic and decision curve analyses validating its predictive accuracy. Thirteen lncRNAs associated with autophagy and cuproptosis were identified through bioinformatics analysis. Lasso regression narrowed this to 3 significant prognostic lncRNAs. Based on median risk scores, patients were classified into high-risk and low-risk groups. Kaplan–Meier survival curves revealed significant differences between these groups (*P* < .01). Through a set of bioinformatics analyses, we identified 13 autophagy- and cuproptosis-related lncRNAs. By Lasso regression, 3 prognostic-related lncRNAs were further selected. We also investigated these 3 lncRNAs in relation to clinicopathologic features. The principal component analysis visually showed differences between the high-risk and low-risk groups.

## 1. Introduction

Head and neck squamous cell carcinoma (HNSC) is a common malignant tumor globally, mainly caused by alcohol, areca nut products, and tobacco exposure.^[1]^ Risk factors include carcinogens, smoking, alcohol, HPV infection, and genetic predisposition. HNSC affects around 900,000 people annually, leading to approximately 500,000 deaths. Late-stage diagnosis is common, resulting in poor prognosis.^[2]^ Although treatments like surgery, radiotherapy, and chemotherapy exist, the clinical diversity and lack of early detection lead to 5-year survival rates of <50%.^[3]^

Long noncoding RNAs (lncRNAs), RNA molecules over 200 nucleotides long, have shown potential as biomarkers for diagnosing and treating various diseases.^[4]^ Advances in high-throughput sequencing and bioinformatics have highlighted that abnormal lncRNA expression plays a significant regulatory role in many physiological and pathological processes, including HNSC.^[5]^ Autophagy-dependent cell death, a form of regulated cell death relying on autophagic machinery, is linked to various regulated cell death types, including cuproptosis.^[6]^ Cuproptosis, a new cell death form mediated by lipoylated TCA cycle proteins and closely related to mitochondrial metabolism, has unclear mechanisms in its interaction with autophagy.^[7]^

This study used bioinformatics to identify lncRNAs related to autophagy and cuproptosis that impact HNSC prognosis, aiming to provide new research directions for understanding the association between HNSC and lncRNAs and improving prognosis evaluation for HNSC patients.

## 2. Materials and methods

**Figure 1.**
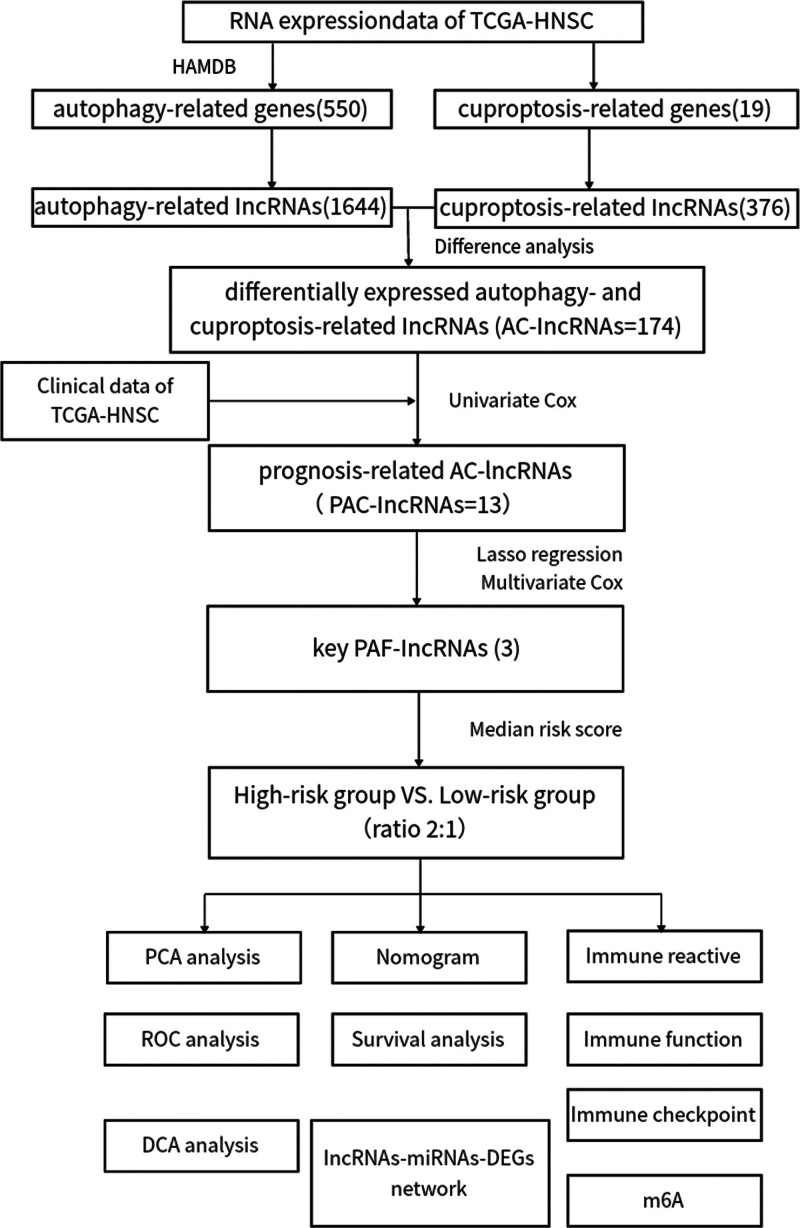
Flowchart: diagram illustrating the step-by-step process of our study, from data collection through to analysis and conclusions.

A flowchart of data collection and analysis is shown in Figure 1. We will elaborate on each step in the next subsections.

### 2.1. Data acquisition

RNA sequencing data were collected from The Cancer Genome Atlas HNSC database for 44 normal patients and 525 tumor patients. We selected 528 patients with complete follow-up information (Table S1, Supplemental Digital Content, http://links.lww.com/MD/N380). A total of 550 autophagic genes were gathered from Human Autophagy Modulator database (Table S2, Supplemental Digital Content, http://links.lww.com/MD/N381). We obtained 19 cuproptosis-related genes (NFE2L2, NLRP3, ATP7B, ATP7A, SLC31A1, FDX1, LIAS, LIPT1, LIPT2, DLD, DLAT, PDHA1, PDHB, MTF1, GLS, CDKN2A, DBT, GCSH, and DLST) from previous studies.^[8]^

### 2.2. Identification of autophagy- and cuproptosis-related lncRNAs

Autophagy-related lncRNAs were identified by Pearson correlation analysis within mRNA and lncRNA expression according to the criteria of |Correlation Coefficient| > 0.4 and a *P*-value < .05. Second, cuproptosis-related lncRNAs were chosen by the same method. Then, we defined both autophagy-related lncRNAs and cuproptosis-related lncRNAs as autophagy- and cuproptosis-related lncRNAs (AC-lncRNAs).

### 2.3. Differential expression and functional analysis

To investigate differentially expressed lncRNAs, we performed differentially expressed analysis with the criteria of |fold change| > 1.5 and *P* < .05. Differentially expressed genes (DEGs) were identified in the same way.

### 2.4. Construction of a prognostic risk model

In order to construct an AC-lncRNAs prognostic model, 499 patients were randomly divided into training and testing sets in a 2:1 ratio, and the prognostic value of each lncRNA in the training set was assessed by univariate Cox regression analysis (*P* < .05), with further screening based on least absolute shrinkage and selection operator regression to avoid overfitting.

In accordance with multivariate Cox analysis, the risk score was computed using the following formula: risk score = coef (lncRNA1) × exp (lncRNA1) + coef (lncRNA2) × exp (lncRNA2)  + ... + coef (lncRNAn) × exp (lncRNAn). The coefficient values (coef) were determined using established methods, as previously reported. The coef represents the coefficient associated with the respective lncRNA, which was calculated using the survival Coxph function of the R package. “exp” denotes the expression level of the corresponding lncRNA. Based on the median risk score, patients with HNSC in the training and testing sets were stratified into high-risk and low-risk groups, respectively. Univariate and multivariate Cox regression analyses were employed to assess the prognostic significance of the risk score and clinical characteristics using the survival R package. The survival status distribution between high-risk and low-risk patients was visualized using the ggplot2 and pheatmap R packages. Principal component analysis was performed to validate the differentiation between the high-risk and low-risk groups. Subsequently, Kaplan–Meier survival analysis was conducted to evaluate the survival disparity between these 2 groups, utilizing the survival and survminer R packages. To assess the sensitivity and specificity of prognosis-related AC-lncRNAs (PAC-lncRNAs) signatures, time-dependent receiver operating characteristic (ROC) curves and multi-factor ROC curves were generated using the time ROC and survival ROC R packages.

### 2.5. lncRNAs–microRNAs–DEGs co-expression network

Potential PAC-lncRNA–targeted microRNAs (miRNAs) were predicted using the StarBase database. Potential DEGs targeting miRNAs were predicted using mirDIP database (minimum score: high). When lncRNA–miRNAs contain common miRNAs, they are selected to construct lncRNA–miRNA–DEG networks. The network was mapped by Cytoscape.

### 2.6. Immunoassay and gene expression

To compare cellular components or cellular immune responses between high-risk and low-risk groups, we used multiple algorithms (TIMER, CIBERSORT, QUANTISEQ, MCPCOUNTER, XCELL, and EPIC)^[9–11]^ based on the characteristics of PAC-lncRNAs. Heat maps were used to demonstrate differences in immune responses under different algorithms.

## 3. Results

### 3.1. Identification of PAC-lncRNAs in HNSC

**Figure 2.**
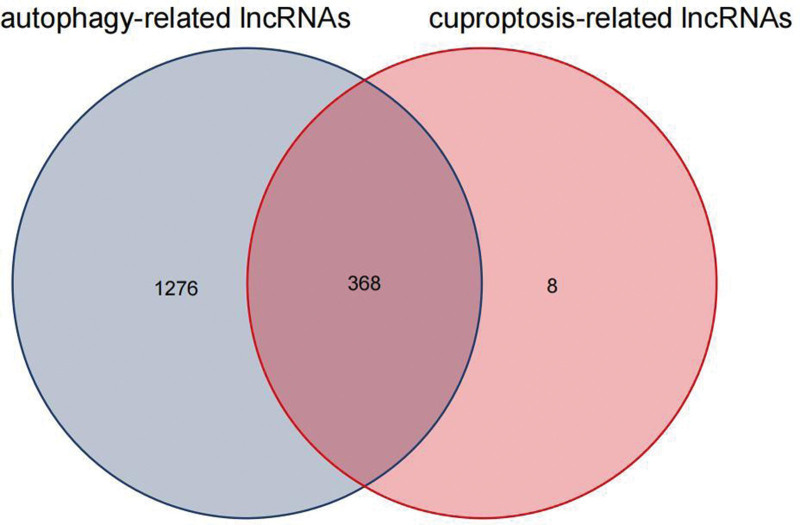
Venn diagram of AC-lncRNAs: shows the overlap between different sets of AC-lncRNAs identified in our study, highlighting common and unique elements.

**Figure 3.**
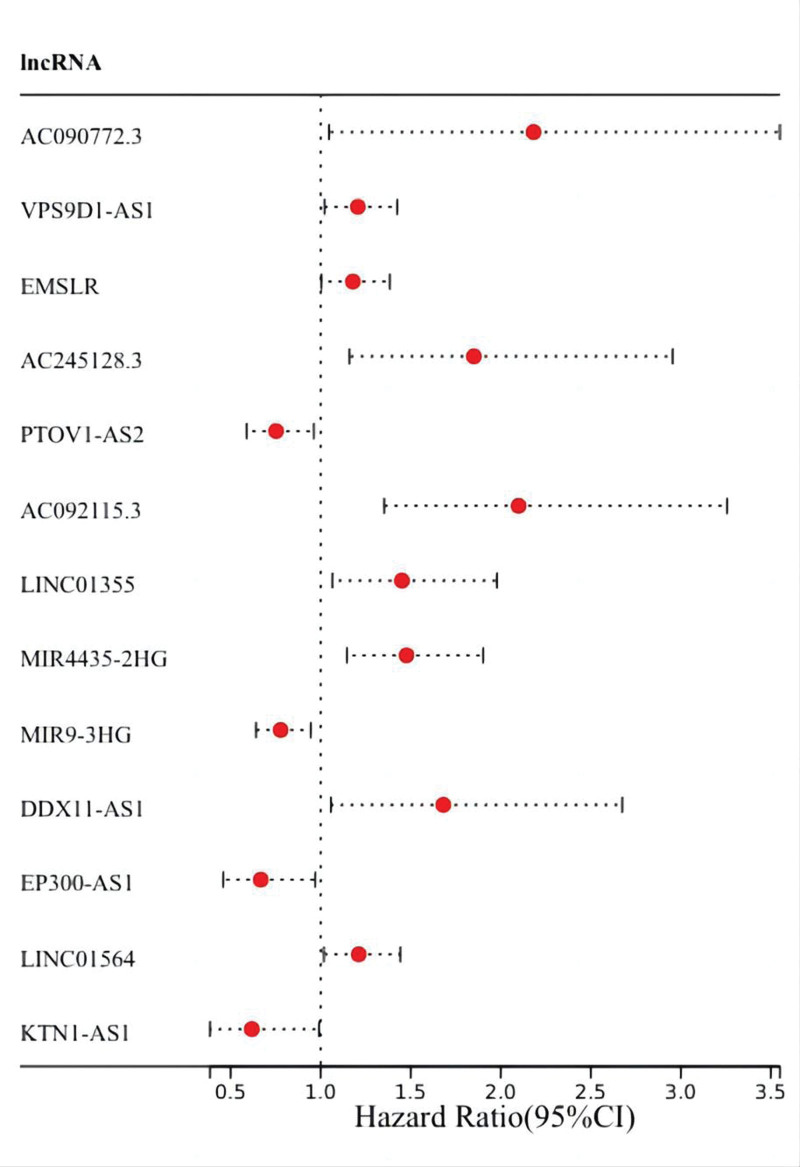
Univariate Cox regression analysis: graphical representation of the univariate Cox regression analysis identifying potential prognostic AC-lncRNAs based on survival data.

**Figure 4.**
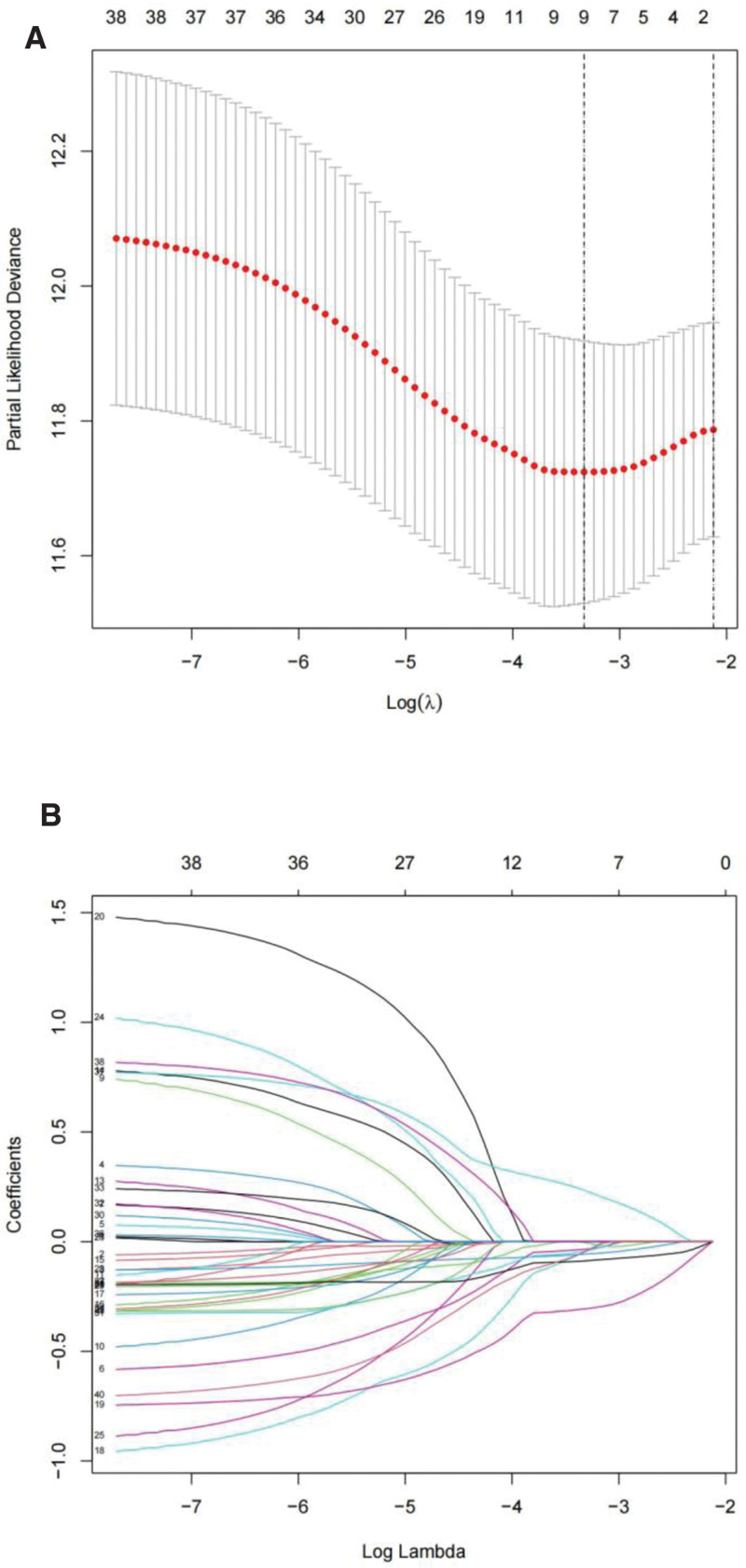
Lasso regression: plots showing the selection of variables for the prognostic model using Lasso regression, illustrating coefficient shrinkage and variable selection.

Firstly, 1644 autophagy-related lncRNAs were identified (Table S3, Supplemental Digital Content, http://links.lww.com/MD/N382). Second, we screened 376 cuproptosis-related lncRNAs by using the same method (Table S4, Supplemental Digital Content, http://links.lww.com/MD/N383). Finally, we got 368 AC-lncRNAs (Fig. 2). By differential analysis, we obtained 174 differentially expressed AC-lncRNAs and 3 DEGs (up-regulation: CDKN2A and GLS; down-regulation: PDHB). Through the univariate Cox analysis, 13 PAC-lncRNAs were initially identified (Fig. 3). Subsequently, a least absolute shrinkage and selection operator regression analysis was conducted to narrow down the selection to 3 key PAC-lncRNAs (Fig. 4). These were AC090772.3, DDX11-AS1, and LINC01315.

### 3.2. Survival analysis and multivariate analysis

**Figure 5.**
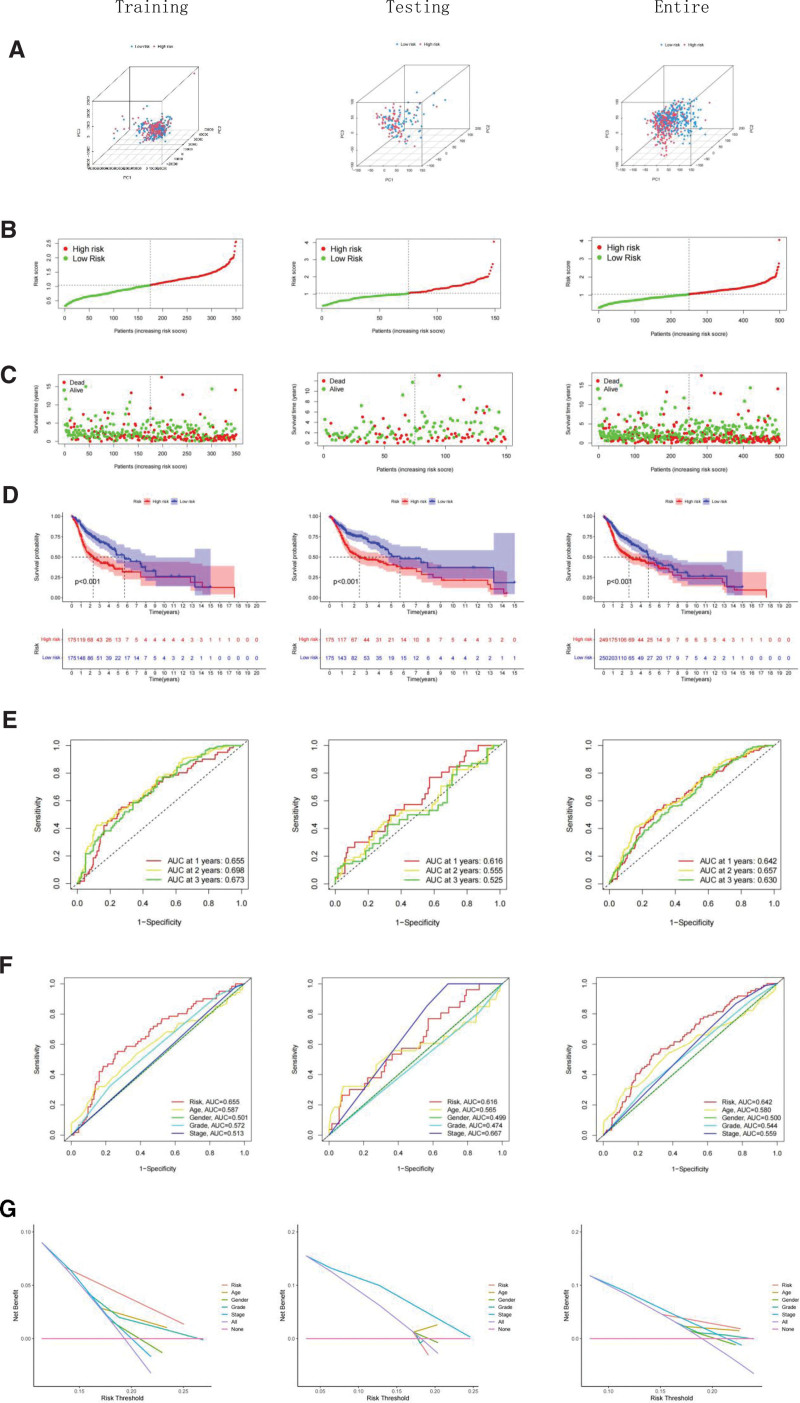
Validation of the prognostic risk model in HNSC: (A) PCA plot demonstrating the distinct clustering of high-risk and low-risk patients according to the prognostic model. (B) Line graph showing the risk score distribution and threshold for classifying patients into high or low risk across different datasets. (C) Scatter plot depicting survival status and risk score across the study cohorts. (D) Kaplan–Meier curves comparing overall survival between high-risk and low-risk groups. (E) Time-dependent ROC curves for 1, 2, and 3 years forecasting the accuracy of the risk model in survival prediction. (F) ROC curve comparison highlighting the superior predictive performance of our risk model against other clinical features. (G) Decision curve analysis (DCA) showing the clinical utility of our model compared to other features.

To evaluate the accuracy of stratification, we conducted several analyses, including principal component analysis (Fig. 5A), risk score analysis (Fig. 5B), and survival status analysis (Fig. 5C). The findings indicated that the risk scores effectively differentiated high-risk patients from low-risk groups in the The Cancer Genome Atlas training, testing, and overall datasets. Kaplan–Meier survival analyses revealed significantly lower survival rates in the high-risk group compared to the low-risk group in both the training, testing, and overall datasets (*P* < .001, Fig. 5D). Additionally, the ROC curves demonstrated that the area under the curve for 1-, 2-, and 3-year survival was consistently >0.6 (Fig. 5E). When compared to traditional clinicopathological features, ROC with decision curve analysis yielded more accurate results in predicting HNSC prognosis (Fig. 5F and G). These results suggest that the risk models based on PAC-lncRNAs exhibit strong predictive power and accurate stratification for HNSC patients.

### 3.3. Independent value of PAC-lncRNAs signature

**Figure 6.**
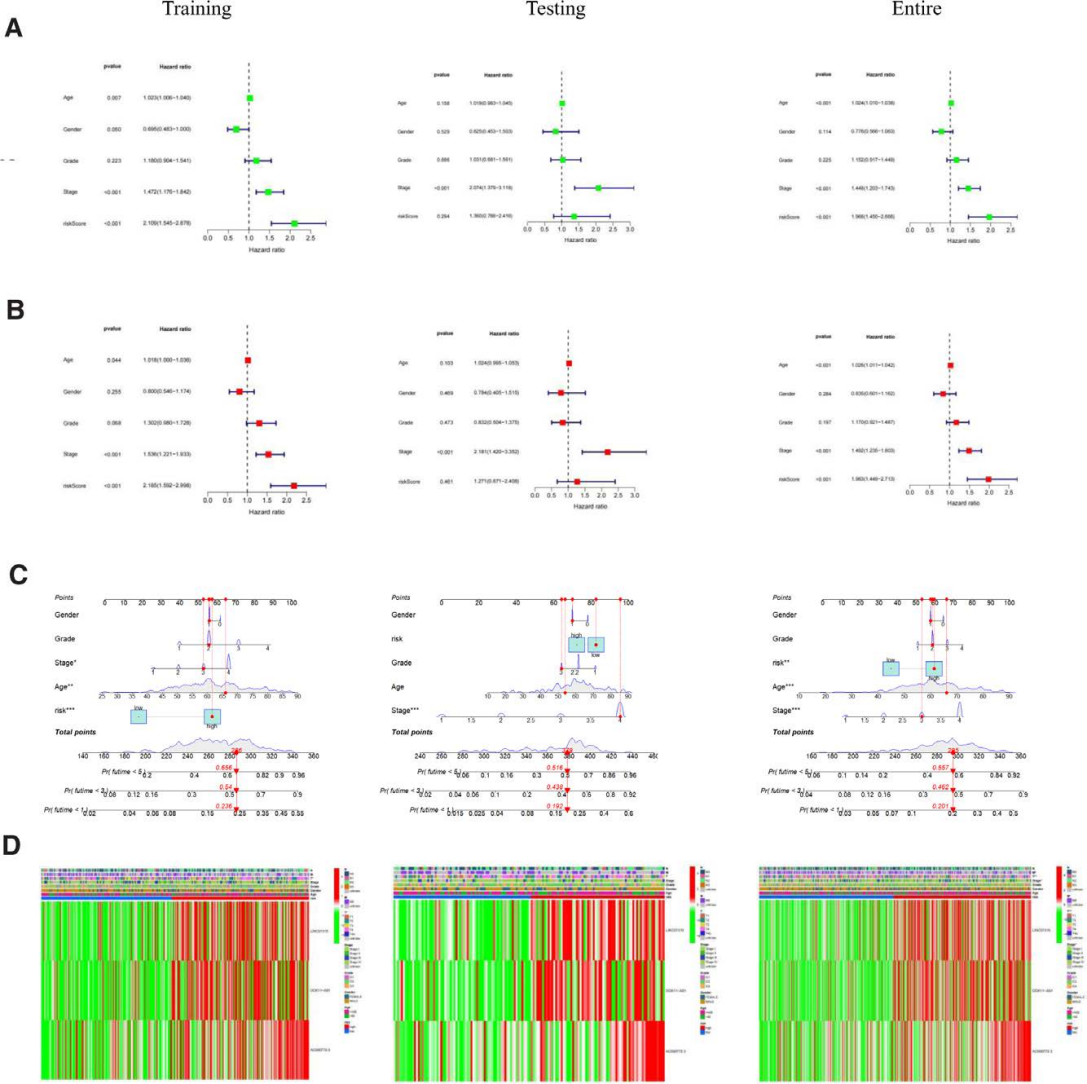
Independent prognostic value of PAC-lncRNAs signature: (A) univariate Cox regression analysis validating the prognostic significance of PAC-lncRNAs across datasets. (B) Multivariate Cox regression analysis confirming the independence of the PAC-lncRNAs signature from other clinical variables. (C) Prognostic nomogram integrating the risk score with clinicopathological factors for predicting survival at 1, 3, and 5 years. (D) Heatmap showing the expression pattern of PAC-lncRNAs across the patient cohorts and its association with clinical features.

To evaluate if the risk score acts as an independent prognostic indicator for patients with HNSC, we carried out both univariate and multivariate Cox regression analyses focusing on clinical characteristics and the risk score. The findings revealed a significant correlation between the risk scores and HNSC across the training, testing, and overall datasets (Fig. 6A and B). Following this, we employed clinicopathological features such as age, gender, grade, stage, and risk score to construct column line plots aimed at predicting the prognosis of HNSC patients (Fig. 6C). Additionally, we created heat maps to display the relationship between cuproptosis-related lncRNAs prognostic factors and clinicopathological features (Fig. 6D).

### 3.4. lncRNAs–miRNAs–DEGs co-expression network

**Figure 7.**
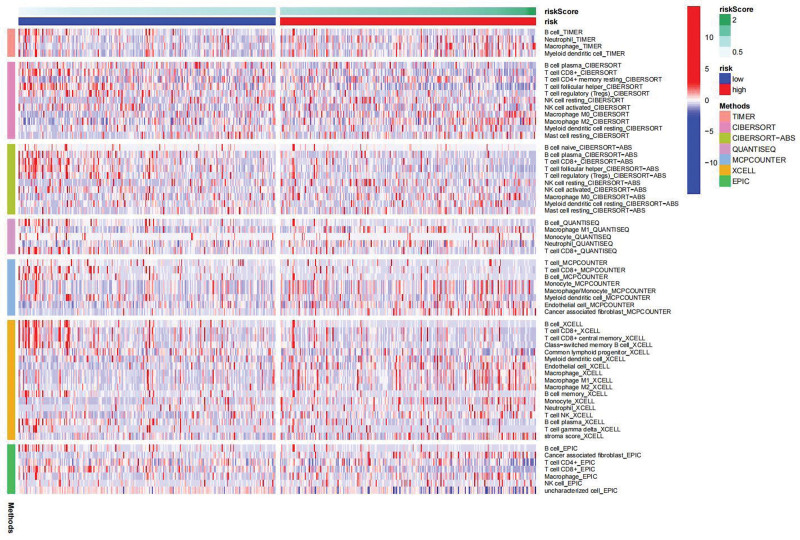
Immune reactive heatmap: heatmap displaying the immune activity profiles across samples, illustrating variations in immune response.

Using the previously obtained PAC-lncRNAs and DEGs as relevant targets, we searched both StarBase and mirDIP databases and identified 239 miRNAs associated with lncRNAs in StarBase and 2145 miRNAs associated with lncRNAs in mirDIP. When we compared miRNAs selected from these 2 databases, we found a common intersection of 206 miRNAs. We forecasted the interactions among PAC-lncRNAs, miRNAs, and DEGs by building co-expression networks. These networks are intended to predict the function of lncRNAs, DEGs, and miRNAs; and their regulatory relationships both upstream and downstream (Fig. 7).

### 3.5. Immunity and gene expression

**Figure 8.**
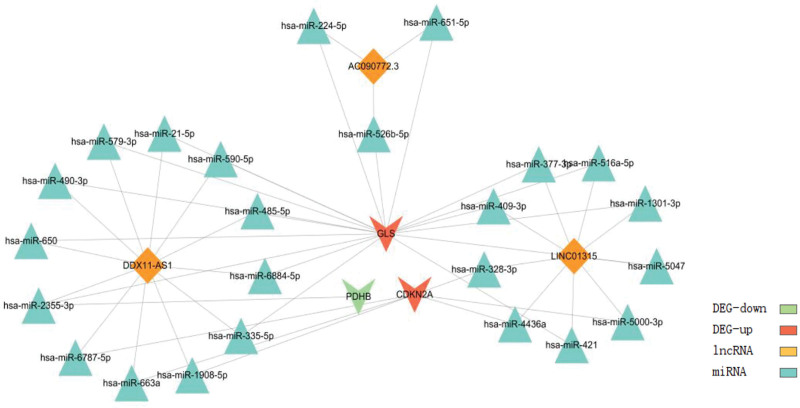
lncRNAs–miRNAs–DEGs network: a network diagram showing the interactions between lncRNAs, miRNAs, and differentially expressed genes (DEGs) identified in our study.

**Figure 9.**
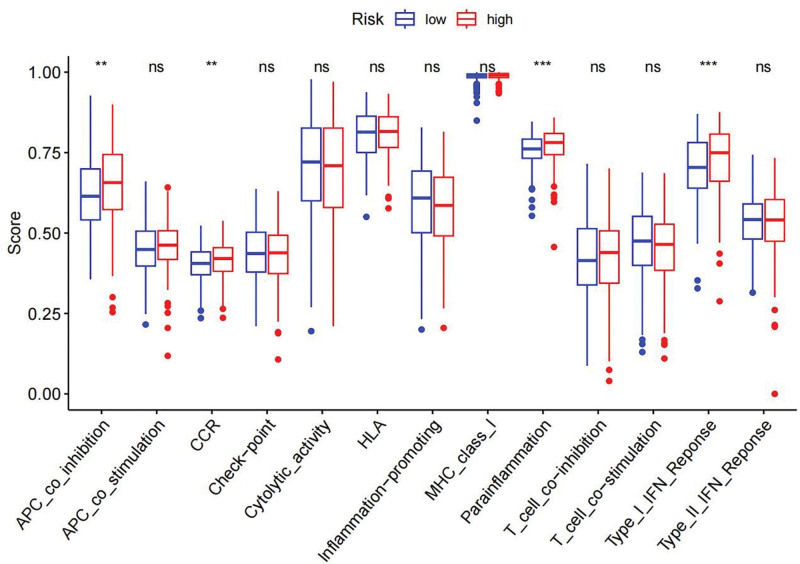
Immune function differential analysis: bar charts and graphs depicting the differences in immune function between different groups based on our analysis.

**Figure 10.**
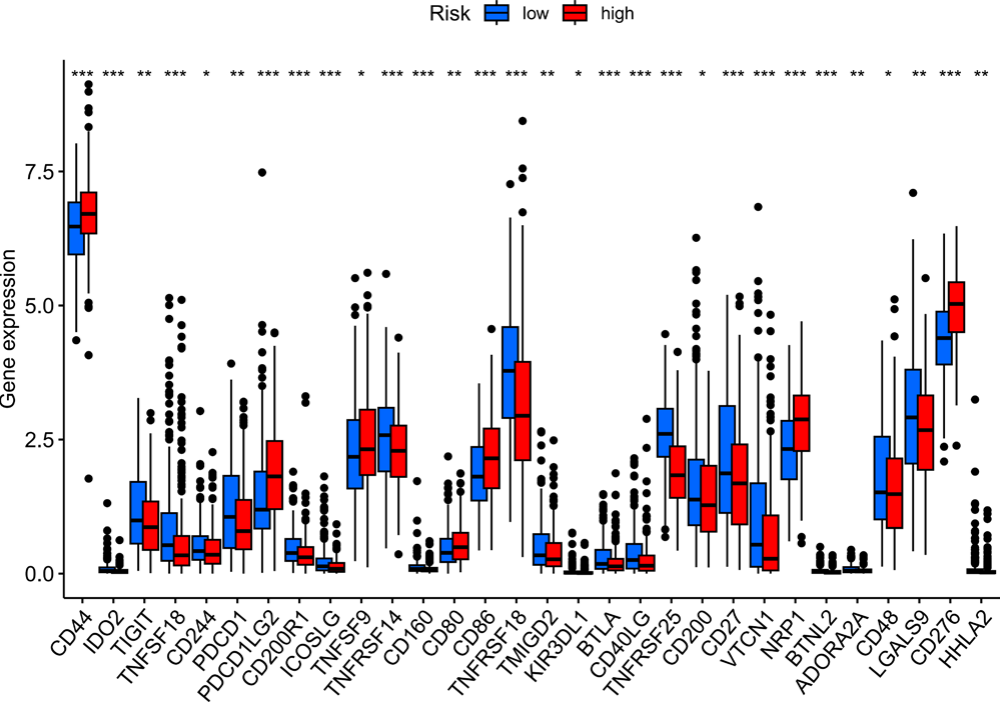
Immune checkpoint analysis: visualization of the expression levels of key immune checkpoints in the context of our study, highlighting potential therapeutic targets. A single asterisk (*) indicates a *P*-value <.05. Two asterisks (**) denote a *P*-value <.01. Three asterisks (***) represent a *P*-value <.001.

**Figure 11.**
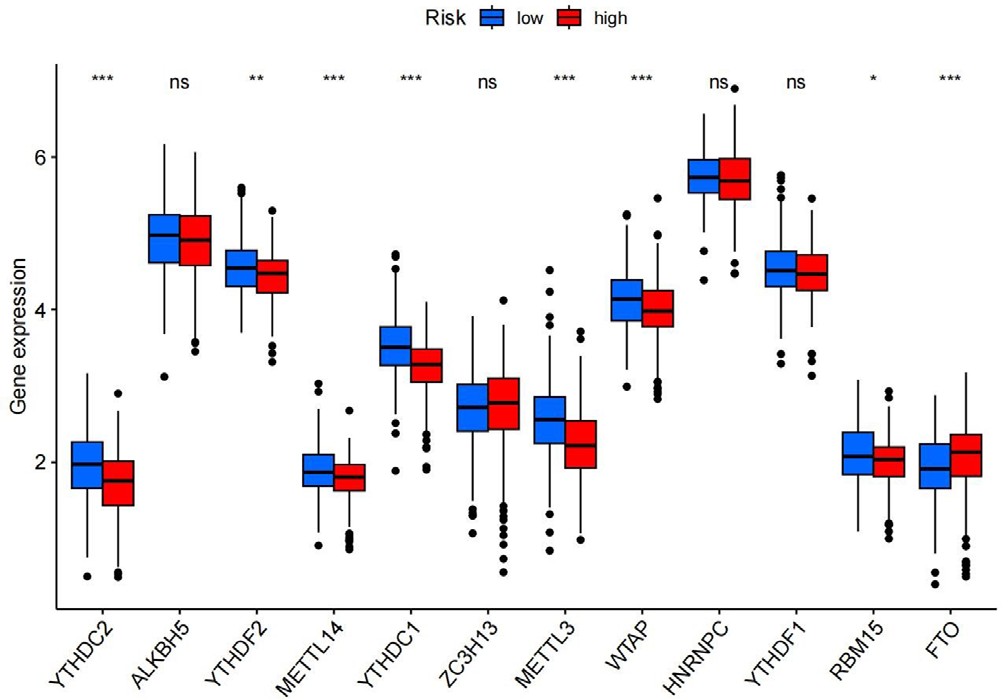
m6A analysis: charts and graphs presenting the analysis of m6A modification patterns in our dataset, exploring their implications for disease prognosis.

Immune response heatmaps were created using these different algorithms (Fig. 8). The correlation analysis revealed notable differences in APC co-inhibition, CCR, MHC class I and Type I IFN response between the high-risk and low-risk groups (Fig. 9). Additionally, immune checkpoints exhibited varying levels of TNFRSF18, CD44, and TNFSF18 (Fig. 10). Considering the significance of checkpoint inhibitor-based immunotherapy, further analysis indicated substantial differences in the levels of YTHDC2, YTHDC1, and WTAP (Fig. 11).

## 4. Discussion

Increasing evidence indicates that the overexpression of DDX11-AS1 plays a dominant role in malignant tumors, showing carcinogenic activity by promoting cancer cell proliferation, migration, invasion, and inhibiting apoptosis.^[12]^

In a study,^[13]^ osteosarcoma cells exhibited significantly increased levels of DDX11-AS1. Experiments showed that reducing DDX11-AS1 hindered cell growth, spread, and the epithelial–mesenchymal transition (EMT) in these cells. Further analysis revealed that DDX11-AS1 regulates DDX11 by sponging miR-873-5p, a process integral to osteosarcoma^[14]^ development. Conversely, elevating miR-873-5p levels curbed cell growth, spread, and EMT. It was also discovered that DDX11-AS1 stabilizes DDX11 mRNA by interacting with IGF2BP2. Additional tests demonstrated that overexpressing DDX11 could counteract the effects of reduced DDX11-AS1 on osteosarcoma progression. In summary, DDX11-AS1 advances osteosarcoma by stabilizing DDX11 expression.

Another study^[15]^ found high levels of DDX11-AS1 in ESCC cells and tissues, correlating with poor patient prognosis. DDX11-AS1 was shown to enhance cell proliferation, migration, invasion, and EMT in vitro. It appears to act as a ceRNA, sponging miR-30d-5p to increase SNAI1 and ZEB2 expression. Additionally, DDX11-AS1 overexpression may activate the Wnt/β-catenin signaling pathway by targeting miR-30d-5p. Overall, these findings suggest DDX11-AS1 as a key EMT-related lncRNA in ESCC progression and a potential therapeutic target, working through miR-30d-5p to regulate SNAI1/ZEB2 and the Wnt/β-catenin pathway. In our study, the appearance of miR-30d-5p undoubtedly enhances the accuracy of our predictions. However, the specific pathway through which miR-30d-5p and DDX11-AS1 influence HNSC expression in tissues remains unexplored. Therefore, we plan to conduct experiments to investigate the specific expression pathways of these molecules in HNSC populations.^[16]^

The antitumor drug paclitaxel (PTX) has been established as an effective treatment for various cancers.^[17]^ However, the resistance of tumors to PTX significantly impacts its clinical efficacy.^[18]^ Zhang S^[19]^ collected tissue samples from esophageal cancer (EC) patients and conducted measurements on a range of lncRNAs, including DDX11-AS1. DDX11-AS1, which had high expression in EC tissues, was studied for its impact on PTX resistance in EC cells by creating PTX-resistant EC cell lines. The role of DDX11-AS1 in the growth of PTX-inhibited tumors was confirmed through a tumor formation assay in nude mice. Research has shown that reducing the levels of the long noncoding RNA DDX11-AS1 in esophageal cancer cells can lessen their resistance to paclitaxel by inhibiting TAF1/TOP2A. Another study^[20]^ also supports this finding. It explored how DDX11-AS1 affects resistance to PTX in lung adenocarcinoma. The study measured DDX11-AS1 expression using quantitative real-time PCR and assessed DNA damage-related protein expression via Western blot. The findings revealed that DDX11-AS1 is highly expressed in lung adenocarcinoma, contributing to increased cell proliferation and PTX resistance while inhibiting cell apoptosis. Additionally, alterations in DDX11-AS1 expression influenced DNA damage repair capabilities.

The impact of LINC01315 on various tumor types has been widely confirmed.^[21–23]^ However, its role in different cancers is debated. In contrast, LINC01315 shows notably higher expression levels in thyroid^[24]^ and colorectal carcinoma (CRC) ^[25]^ compared to noncancerous tissues or normal cells. Yan X^[26]^ conducted in vitro cell experiments to further investigate LINC01315. The study found that upregulating LINC01315, which was reversed by introducing LINC01315 interference RNA, significantly hindered key cellular processes in triple-negative breast cancer. This aligns with LINC01315’s function in colorectal and papillary thyroid cancers, suggesting its role as a tumor enhancer in triple-negative breast cancer.

The Wnt/β-catenin signaling pathway, which we mentioned earlier, also plays an important role in CRC.^[27,28]^ To verify that the increase in invasion and EMT of CRC cells by LINC01315 was β-catenin dependent, LINC01315-silenced CRC cells were transfected with a pcDNA3.1-β-catenin plasmid to restore β-catenin expression. This restoration reversed the suppression of proliferation, clonogenic ability, migration, and invasiveness caused by LINC01315 inhibition. Additionally, LINC01315 inhibition decreased N-cadherin levels and increased E-cadherin expression, but β-catenin overexpression counteracted this trend. These findings indicate LINC01315’s pro-oncogenic functions in CRC are reliant on Wnt/β-catenin signaling.

## 5. Conclusions

Cuproptosis-related cell death, a novel form of cell demise, offers potential new paths for tumor treatment. Yet, key challenges like the link between cuproptosis, autophagy, and the host immune response demand urgent attention. This study built a new model of AC-lncRNAs to identify biomarkers for predicting HNSC prognosis, which could guide treatment strategies. However, due to limited clinical data, this prognostic model needs more validation.

## Acknowledgments

We acknowledge TCGA database for providing their platforms and contributors for uploading meaningful datasets.

## Author contributions

**Conceptualization:** Yan Li.

**Funding acquisition:** Yan Li.

**Methodology:** Yan Li.

**Supervision:** Qianrong Li.

**Validation:** Qianrong Li.

**Writing – original draft:** Yao Xiao, Qianrong Li.

**Writing – review & editing:** Yao Xiao.

## Supplementary Material
